# Glucometer Usability for 65+ Type 2 Diabetes Patients: Insights on Physical and Cognitive Issues

**DOI:** 10.3390/s22166202

**Published:** 2022-08-18

**Authors:** Maria Pinelli, Emanuele Lettieri, Andrea Boaretto, Carlo Casile, Giuseppe Citro, Bernardino Zazzaro, Adriana Ravazzoni

**Affiliations:** 1 Department of Management, Economics and Industrial Engineering, Politecnico di Milano, Via Lambruschini 4/B, 20156 Milan, Italy; 2 Personalive S.r.l., Via Durando 38, 20158 Milan, Italy; 3 Azienda Ospedaliera Papardo, Contrada Papardo, 98158 Messina, Italy; 4 ASP Basilicata, Via Torraca 2, 85100 Potenza, Italy; 5 Presidio Ospedaliero Umberto I° UOS Endocrinologia, Via Testaferrata 1, 96100 Siracusa, Italy

**Keywords:** self-monitoring of blood glucose (SMBG), diabetes, glucose monitoring, glucometer, usability

## Abstract

**Background**: Self-monitoring of blood glucose (SMBG) is of paramount relevance for type 2 diabetes mellitus (T2DM) patients. However, past evidence shows that there are physical and cognitive issues that might limit the usage of glucometers by T2DM patients aged 65 years and over. **Objective**: Our aim was to investigate the physical and cognitive issues related to the usage of glucometers by T2DM patients aged 65 years and over. **Materials and Methods**: The extant literature was analysed to define an original framework showing the logical nexus between physical and cognitive issues and quality of life. Then we collected evidence addressing the specific case of the Accu-Chek^®^ Instant glucometer produced by Roche Diabetes Care GmbH, which implements new features claiming to improve usability. We conducted 30 interviews with T2DM patients aged 65 years and over, three interviews with senior nurses, and a focus group with three senior physicians and three senior nurses. **Results**: From the interviews, both patients and nurses declared that they were generally satisfied with the Accu-Chek^®^ Instant glucometer’s characteristics. In the focus group, the results were commented on and, in the light of some diverging answers, improvements have been set up for future implementation. **Conclusions**: Our study produces evidence and future suggestions about the usage of glucometers by type 2 diabetes patients aged 65 years and over.

## 1. Introduction

Self-monitoring of blood glucose (SMBG) remains a cornerstone of diabetes management as it helps diabetes patients achieve and maintain glycaemic control. Past research pointed out that proper usage of the glucometer positively affects patients’ engagement and adherence to the treatment, resulting in an improvement in their quality of life [1,2].

However, a large body of past evidence (e.g., [3,4,5]) confirmed the distresses experienced by patients of 65 years and over while using devices and mobile applications for monitoring their health, as well as the relevance of investigating the physical and cognitive barriers that might stretch the distance between the elderly and technology.

This study aims at gathering novel evidence about the physical and cognitive issues related to the usage of glucometers for SMBG by type 2 patients aged 65 years and over. The first goal of the study was to review the literature and understand how these issues are related to patients’ quality of life and how SMBG features can solve some of the T2DM patients’ issues. Then, to validate the results of the literature review, the study investigated glucometer usability for T2DM patients aged 65 years and over. The validation of the results was carried out referring to one specific glucometer: the Accu-Chek^®^ Instant glucometer produced by Roche Diabetes Care GmbH. This study does not aim to carry out a comparative study of different glucometers, but it aims at gathering novel evidence about the design features a glucometer must have to engage elderly users. The Accu-Chek^®^ Instant glucometer was used to corroborate the results of the literature review since it is particularly interesting for this study. This is because the manufacturer claims to have new features such as a backlit display, a Target Range Indicator (TRI), and larger and automatically ejected stripes that might benefit patients aged 65 years and over by addressing their potential physical and cognitive problems.

## 2. Materials and Methods

The main goal of the study is to collect evidence about physical and cognitive problems that T2DM patients encounter when using glucometers for SMBG to further the ongoing debate amongst the diabetes scientific community and the manufacturers. For this reason, we decided to carry out a twofold analysis:

- A literature review to gather insights about physical and cognitive issues encountered by T2DM patients in SBGM;
- Interviews and one focus group to validate the results of the literature review.

The following two sub-sections explain the methodology used for both aims.

### 2.1. Literature Review

To analyse the literature, the investigation proceeded from general to particular situations to evaluate the main barriers faced by patients while measuring glycemia, particularly in terms of how to interpret the results and how to act on them.

The results of the literature review were then summarised (i) in a framework connecting the issues in the usage of glucometers with patients’ quality of life and (ii) in a table showing the SBGM characteristics that can solve T2DM patients’ challenges.

Having combined the results of the two mentioned analyses, a questionnaire was drafted to validate, through several interviews, what emerged from the literature and to investigate further needs and problems of patients currently employing the Accu-Chek^®^ Instant SMBG system.

### 2.2. Interviews and Focus Group

Findings from past studies were discussed, first, through in-depth interviews (IDIs) with three senior nurses and, second, within one focus group consisting of three senior physicians and three senior nurses whose patients had gained experience of using the Accu-Chek^®^ Instant glucometer.

Given the explorative nature of this study, we agreed that interviews could offer an opportunity to deep-dive into the research topic [6] and gather new evidence about the physical and cognitive issues concerning SMBG in T2DM patients aged 65 years and above.

Data collection has been organized into three sequential rounds.

First, we video interviewed 30 T2DM over-65-year-old Italian patients who had been using the Accu-Chek^®^ Instant glucometer for at least one month to avoid early-stage opinions. Interviews were carried out in the timespan from December 2021 to January 2022.

In Table 1, the main characteristics of the interviewees are represented.

The sample, aged between 65 and 80 years old, is well-balanced in terms of gender; 40% were women and the remaining 60% were men. It is not representative of all of the Italian population. Interviewees come from the only Italian regions in which the Accu-Chek^®^ Instant glucometer was diffused at the time of the interviews thus representing its “early adopters”. These regions are: Sicily, Piedmont and Basilicata.

The interviews lasted an average of 20 min and have been recorded with the explicit consent of the patients. Two of the authors ran all the interviews. While one conducted the interviews as the main voice, the other took notes and checked that all questions were addressed thus freeing the former interviewer from this responsibility. The interview was organized into three blocks. First, getting the profile of the patient; second, investigating her/his familiarity with the glucometer, and, third, understanding the usability of the Accu-Chek^®^ Instant glucometer. Even if the sample of interviews is of moderate size (i.e., 30 patients), we decided to implement a 5-point Likert scale, ranging from “strongly disagree” to “strongly agree”, to measure the level of agreement/disagreement on the usability of the new glucometers’ features.

Second, the insights emerging from the interviews were discussed with three senior nurses who have significant experience of SMBG in T2DM patients aged 65 years and over. They helped us to make sense of the richness of patients’ opinions and to organize the third round of data collection.

Third, we organized one focus group involving three senior physicians and three senior nurses (others than those involved in the previous phase). The focus group was managed virtually via Google Meet because of the restrictions on vis-à-vis meetings as a consequence of the pandemic flu.

Before starting the data collection, we informed Roche Diabetes Care Italy S.p.A. about this study, and we asked them for any available information about the Accu-Chek^®^ Instant glucometer. Roche Diabetes Care Italy S.p.A. shared with us, upon NDA, an internal report about the device and the results of international research conducted in five countries (Germany, India, United Kingdom, Mexico, and United Arab Emirates) about the ease of use of the new Accu-Chek^®^ Instant glucometer before using it in a routine way [7].

## 3. Results

In this section, the main findings from the literature review and the three rounds of data collection are reported briefly.

### 3.1. Findings from the Literature Review

Regarding physical barriers to technology, past studies pointed out that visual impairment and haptic deterioration are the main issues faced by patients aged 65 years and over [8,9]. As result, they feel uncomfortable when they deal with buttons that are either too small or rubbery; they prefer metallic buttons which click when pressed so that they can better understand whether they pushed them or not. Menus with too many options might appear as unnecessary or excessively complicated. Small devices or screens might be uncomfortable for the user and even hard to read [8,10,11].

Solutions to these physical issues are numerous. Visual impairment issues might be lessened through the use of larger displays that show large enough font-size text. Customizable font size is a feature that is appreciated by users who can enlarge it as needed. The high and comfortable colour contrast between the foreground and the background is helpful to improve readability.

Concerning the haptic problem, a device should be big enough to be grabbed and held comfortably by the user and made with non-slippery and easy-to-clean materials. Buttons, where present, should be square, made of metal, raised, and not too sensitive to avoid the risk of pressing them accidentally [12,13,14].

Cognitive issues deserve special attention, too. They can worsen elderly people’s living conditions [12,13]: fine movements, motor control, and cognitive functions, such as perception, decline with age. Memory loss leads to forgetting some steps even when performing simple tasks and many difficulties are encountered when managing different activities simultaneously. It might be difficult for patients aged 65 and over to understand a large amount of information at once. No less important is the problem of speed of execution that results in the inability to carry out operations quickly. Finally, difficulties might also appear in the decision-making process, in solving problems, planning, storing, and managing new information [15].

Past studies offer solutions to address cognitive issues. Guided operations or longer screen-dimming time would allow enough time for cognitive processing and executing operations. Embedding auditory and tactile feedback as well as inserting familiar features might increase users’ confidence in the usage of the device and improve the degree of autonomy. Dealing with procedures or activities like others previously performed might help ease the user experience. The appliances must be more intuitive and self-explanatory to increase usability [16,17,18].

Zooming in on the case of SMBG in patients aged 65 years and over, especially those with type 2 diabetes mellitus (T2DM), two main issues have been identified by past studies [19,20]. On the one hand, the difficulty in interpreting results and, on the other hand, the limited literacy about how to act upon results.

While T2DM patients might think that identifying low, high, and in-range glucose values is relatively easy, evidence shows that their ability to interpret blood glucose (BG) is still to be improved [19] because many of them are not able to interpret the results displayed on their meter and, as result, they take no action or make any self-care adjustments for out-of-range results [20].

The misinterpretation of results leads to a lack of corrective actions on the results obtained, low compliance to physicians’ recommended deadlines for follow-up visits, and a reduced number of daily or weekly measurements performed [20]. A large-sized international study on SMBG pointed out that treatment adherence was around 24% for T2DM patients [21]; this highlights the need for designing elderly-friendly glucometers to meet users’ needs.

An example is given by the capability to improve glycaemic control when the elderly switch to a blood glucose measurement based on a TRI with different colours from traditional blood glucometers (BGMs) without a colour scale [19]. The TRI can be individualized for the patients’ needs according to the therapeutic range in agreement with the health care professional (HCP). By using a colour scale, patients can better interpret results, classify BG readings as low, high or in range, and make decisions accordingly [19]. The colour scale allows the definition of a glucose result as either “near low” or “near high” by glancing in the direction of below or above range segments, allowing patients to either take action before their score would become too high/too low or reflect on the reasons that generated “near low”/“near high” results [22,23]. The introduction of the colour system improves patients’ feeling of being safe and in control because they are alerted when results are near high/low values [22,24,25].

A recent randomized controlled study [26] found that the usage of BGMs with Colour Range Indicator (CRI) reduces glycated haemoglobin (HbA1C)—HbA1C is a gold standard blood test for patients with diabetes that measures the proportion of glycated haemoglobin which is in correlation with the blood glucose level over the past 3 months. Other studies proved that the use of CRI improves the management of diabetes [20,22,23,25].

**Figure 1:**
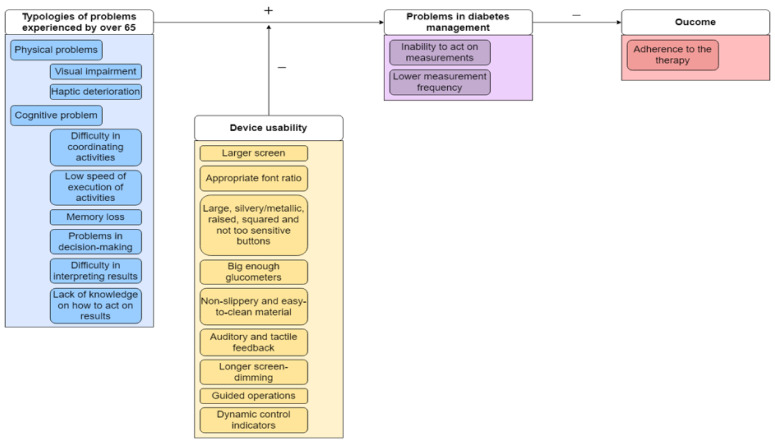
Logical connections about quality of life and issues in the usage of glucometers for SMBG.

Figure 1 offers an immediate visual representation of the results of the literature regarding the causality nexus between the issues that emerged from the extant literature and quality of life as the most impacted outcome.

Table 2 shows what features of a glucometer might ameliorate the issues identified in the literature review.

In summary, past studies argued that there was still an unmet need of providing T2DM patients with easy-to-use devices that can increase adherence to the prescribed treatment.

### 3.2. Findings from Interviews with Patients

Coherently to the findings that emerged from the literature (Figure 1), we wanted to investigate if some features could help patients aged 65 years and over to improve the management of their disease as a result of a simpler and easier SMBG practice. As explained in the materials and methods section, we carried out interviews with patients using the Accu-Chek^®^ Instant glucometer, since it is particularly appropriate for our study. Its characteristics are linked to the results of the literature review. The backlit display increases the contrast between the screen foreground and background thus enhancing readability and helping with the problem of visual impairment. An automatic test strip ejector and larger dosing area make handling easier, addressing the problem of haptic deterioration. Finally, the colour range scale improves the measurement understanding, which tackles the cognitive problem of difficulty in interpreting the results.

In the following section, the results of the interviews are organized in Table 3 and then commented on. We used a standard questionnaire composed of six questions (visible in the first column) and patients were invited to answer using a range from one (disagree strongly) to five (agree strongly).

Regarding the first and the second questions, the majority of patients thought that the backlit display made it easier to read the results on the screen and that the wide dosing area made it easier to apply the blood at the end of the strip.

In answering the third question, each patient agreed on the increased level of hygiene and practicality that the automatic ejection of the strip guarantees.

Regarding the TRI feature addressed in the fourth question, half of the sample used the colour scale and agreed that it made it easier to understand or confirm the result and to carry out corrective actions, while the other patients were not even aware of the presence of the colour scale, and so, did not use it

In addition, regarding the fifth question, it has been found that for only half of the sample, the effort required to learn how to use the new device was low because of similarities to previous devices. Some patients highlighted that the device is easy to use but it requires HCP support and training.

The interviews also showed that the most significant concern about the Accu-Chek^®^ Instant glucometer is the use of the pricking pen since elderly people’s thick skin is often hard to prick.

Lastly, a direct relation emerged between the patients’ technological literacy and their appreciation of the glucometer app features; those who are more accustomed to the use of digital tools, as well as caregivers, feel calmer having results automatically stored in the app.

To conclude with the final question, most of the patients argued that they would recommend this glucometer to senior patients because the new features improve its usability.

### 3.3. Findings from Interviews with Nurses

Nurses were asked about the selection of glucometers for their patients, and they declared using specific parameters connected to the characteristics of the patients, which is totally in line with the literature results. The characteristics they take into consideration are age, ability to use digital devices, physical problems, sensitivity and visibility.

One nurse focused on connectivity features, and she considered the Accu-Chek^®^ Instant glucometer more appropriate for “younger” patients without mobility or visibility problems. The other two nurses, instead, appreciated the essential and intuitive design, judging the “easy-to-use” and “easy-to-learn” features to be very suitable for T2DM over-65 patients.

During the interview, all nurses stressed the importance of delivery of the device and training sessions with patients, starting from finger pricking to the right use of glycaemic value measured in their daily life. The lancing device is regarded as a very important component, especially for those people having difficulties handling devices. The nurses declared that the lancing device used with Accu-Chek^®^ Instant is particularly easy to use and effective, being able to perforate the skin at the first attempt without causing excessive discomfort. Nevertheless, in some cases, the nurses did not provide the patients with any educational materials during the training, due to their age, but probably these subjects especially need more support with memory loss or attention deficit. This situation can be connected with the answers obtained from the patients who did not appreciate the colour scale; it could be better indicated and explained in this support material.

Finally, they claimed that so far none of their over-65 patients complained about the Accu-Chek^®^ Instant glucometer and none of them asked to change the glucometer.

### 3.4. Findings from the Focus Group

The focus group dealt with three main topics.

First, collecting professionals’ opinions on the findings that emerged from the literature and the interviews and their professional experience. Second, gathering their expert opinions about glucometers for over-65-year-old T2DM patients. Third, collecting their feedback on the Accu-Chek^®^ Instant glucometer.

Participants unanimously confirmed the main findings crystallized from the literature review and the logical nexus between the physical and cognitive issues and quality of life. They reinforced the need to facilitate SMBG in T2DM patients over the age of 65 to sustain their commitment to being adherent to therapy. The ease of use of glucometers is a key factor and there is still room for improvement.

Concerning the Accu-Chek^®^ Instant glucometer, they pointed out some positive elements. First, T2DM over-65-year-old patients are very different among themselves in terms of skills and technological literacy. In this view, the high level of customizability of parameters enabled by the app was largely appreciated by the professionals. Second, the design of the glucometer echoes other senior-friendly digital tools (e.g., TV remote controllers, smartphones, etc.) thus leveraging “familiarity” to reduce cognitive issues.

Professionals agreed on the need to have “a glucometer that works for everyone” with very easy functionalities that might also be used by less-educated over-65-year-old patients. They pointed out that the Accu-Chek^®^ Instant glucometer has brought improvements in this direction.

Both physicians and nurses shared concerns regarding patients’ lack of awareness about TRI. In their words, this finding points out the need to reinforce the training program for patients. Patients must be fully aware and confident about how to use the glucometer. Therefore, this result needs further research to better identify the gaps and propose an improved training program. A preliminary idea shared within the focus group included the production of video tutorials. Another solution might be matching the information on the colour with a recommended action to be undertaken.

Finally, the participants agreed on the urgency to involve patients in the development of devices. Co-design and co-production of innovation in healthcare [27] must be on the agenda of policy makers and developers. Collecting the “voice” of patients-especially those aged 65 years and above-is necessary to increase the acceptance of these innovations and exploit their potential.

## 4. Discussion

This study provides novel insights to further the ongoing debate about both the physical and cognitive issues that might limit SMBG in T2DM patients over 65 years old. To address the first aim of the paper, related to the literature review, we offer (Figure 1) an original synoptic view of the logical nexus between the physical and cognitive issues and quality of life, as one of the most relevant outcomes that might be affected by a friendly glucometer. With this framework, we can contribute to the literature regarding the SMBG in T2DM patients over 65 years old.

Concerning the specific case of the Accu-Chek^®^ Instant glucometer, interviewed patients pointed out that the backlit display and the automatic strip ejection features are useful and appreciated. Vice versa, mixed opinions emerged about the TRI: the literature demonstrates that the tool itself is useful but probably this feature is not self-explanatory and appropriate training on it should be an important part of the conversation with health care professionals. Target ranges should be discussed to support patients and bring value to their diabetes management. In addition, further studies are requested to ascertain whether the continued and consistent use of the colour-based blood glucose meter could result in improved outcomes for patients. Investigating the case of the Accu-Chek^®^ Instant glucometer, it has been possible to validate the results of the literature and to collect insights that can be applied to glucometers in general.

### Study Limitations

Our findings must be interpreted in light of the limitations of this research. First, this study has a qualitative research design with a moderate sample of T2DM over-65-year-old patients. Second, all data collection refers to three Italian regions. Third, data have been collected only through digital means because of the restrictions due to the pandemic flu.

## 5. Conclusions

Self-monitoring of blood glucose (SMBG) is still a cornerstone of diabetes management for a lot of people with diabetes, mainly type 2, to maintain and improve glycaemic control. This study found, through the literature, a nexus between physical and cognitive barriers to the usage of glucometers for SMBG and quality of life, and it was confirmed by empirical analysis. The interviewees reported that the Accu-Chek^®^ Instant glucometer is easy to use and brings benefits to them. However, it is clear that the key to SMBG success is patients’ engagement and adherence to the treatment. The meaning of a blood glucose value beyond a pure number is crucial and the technical features of devices might be supportive, especially for patients who have a numeracy deficit or a limited ability to remember any information from training sessions. Digital functionalities are also often perceived as appropriate only for younger target groups, but connectivity may not be necessary at the patient level. However, it can support healthcare professionals and caregivers in providing a rapid overview of data and the individualisation of therapeutic settings [28].

### Future Analysis

As outlined in the discussion section, the sample of this research is small, so future works should involve a larger number of patients through extensive survey. Moreover, future studies should collect data in other Italian regions to have a sample that is representative of all of the Italian population. It would be optimal to involve patients from other countries to argue the generalizability of our results. Finally, further works should discuss the opportunity of vis-à-vis data collection to reduce potential biases due to the usage of digital means.

## Figures and Tables

**Table 1:** Sample Characteristics.

Sample Characteristics	
Gender	40% women, 60% men
Age	65–80 years old
Origin	Sicily, Basilicata and Piedmont.

**Table 2:** SMBG features solving T2DM 65+ patients’ issues.

Issue	SMBG Features to Address It
Visual impairment	Larger screen Appropriate font ratio Auditory and tactile feedback
Haptic deterioration	Large, silvery/metallic, raised, squared and not too sensitive buttons Big enough glucometers Non-slippery and easy-to-clean material
Low speed of execution of activities	Longer screen-dimming
Memory loss	Guided operations
Difficulty in coordinating activities	Auditory and tactile feedback
Difficulty in interpreting results	Dynamic control indicators

**Table 3:** Patients’ answers regarding the features of Accu-Chek^®^ Instant glucometer.

*#*	*Questions*	*Agree Strongly*	*Agree*	*Neither Agree or Disagree*	*Disagree*	*Disagree Strongly*	*Total Agreement*	*Mean Grade*
1	I believe the backlit display makes it easy to read the results on the screen	38%	48%	17%	0%	0%	86%	4.1
2	The wide dosing area will make it easy to apply the blood at the end of the strip	34%	45%	17%	3%	0%	79%	4.1
3	The strip ejector makes it easy to remove the strip hygienically	48%	28%	21%	3%	0%	76%	4.2
4	The colour scale (target range indicator) makes it easy to understand the results quickly	17%	38%	10%	3%	31%	55%	3.1
5	I believe it is easy to learn and use without HCP training	45%	34%	17%	3%	0%	79%	4.2
6	I would recommend this glucometer	50%	39%	4%	7%	0%	89%	4.3
